# Association of the modified creatinine index with muscle strength and mortality in patients undergoing hemodialysis

**DOI:** 10.1080/0886022X.2022.2134027

**Published:** 2022-10-17

**Authors:** Rongrong Tian, Liyang Chang, Dan Liu, Fenxia Luo, Ying Zhang, Linghong Cheng, Hongmei Zhang

**Affiliations:** a Department of Blood Purification Centre, Hangzhou TCM Hospital Affiliated to Zhejiang Chinese Medical University, Hangzhou, Zhejiang Province, China; b The Department of Science and Development, The Second Affiliated Hospital of Zhejiang University School of Medicine, Hangzhou, Zhejiang Province, China

**Keywords:** Modified creatinine index, muscle strength, sarcopenia, hemodialysis, mortality

## Abstract

**Background:**

In the updated consensus, low muscle strength overtook the role of low muscle mass, and probable sarcopenia was diagnosed once low muscle strength was detected. Whether the modified creatinine index (mCI) could identify persons with probable sarcopenia who may be at risk of adverse outcomes remains unknown. We aimed to evaluate the association of the mCI with probable sarcopenia and mortality in patients undergoing hemodialysis.

**Methods:**

In the cross-sectional study (*n* = 346), univariate and multivariable logistic regression analyses were performed to study the association of mCI with probable sarcopenia. Modified Quantitative Subjective Global Assessment (MQSGA) was used to evaluate the nutritional status. The performance of the mCI value for identifying probable sarcopenia was analyzed using receiver operating characteristic (ROC) curve analysis. The appropriate cutoff points were determined using Youden’s method. In the longitudinal cohort study composed of an independent hemodialysis cohort (*n* = 218), cox proportional regression models were used to evaluate crude and adjusted hazard ratios and 95% confidence intervals (CIs) of death by mCI and MQSGA.

**Results:**

Cross-sectional results showed that after adjusting for confounders, the association of mCI with low muscle strength remained significant. The area under the curve (AUC) of the mCI to predict probable sarcopenia was 0.804 (95% CI, 0.744–0.863; *p* < 0.001) for men and 0.787 (95% CI, 0.711–0.864; *p* < 0.001) for women. The optimal mCI cutoff values were 21.07 mg/kg/d for men and 19.57 mg/kg/d for women, respectively. Longitudinal results showed that compared with those in the high mCI group, subjects in the low mCI group had a higher risk of death for all causes (adjusted HR, 2.51; 95% CI, 1.16–5.41; *p* = 0.019). Adding the mCI significantly improved the predictive accuracy for death with an increase in C-index from 0.785 to 0.805 (*p* = 0.026) and improved the net reclassification index (38.6%, *p* = 0.021), while adding MQSGA did not.

**Conclusion:**

The mCI is a predictor of muscle strength and survival in hemodialysis patients, and is preferable to the MQSGA for predicting death. Assessment of mCI could provide additional predictive and prognostic information to sarcopenia.

## Introduction

Patients with chronic kidney disease (CKD) are considered to be in a state of decreased muscle protein synthesis and increased protein catabolism, resulting in muscle wasting and a gradual decline in muscle function [1,2]. Therefore, sarcopenia, a syndrome characterized by low muscle mass and function, is highly prevalent in patients undergoing dialysis and is associated with adverse clinical adverse outcomes [3–5]. Therefore, screening for sarcopenia in a timely manner is essential for early interventions [6]. However, measuring muscle mass requires special equipment, which is not always feasible and has drawbacks, such as high cost, radiation exposure, and poor accessibility. Thus, sarcopenia has been undertreated in clinical practice.

The creatinine kinetic modeling (CKM)-derived creatinine index was developed as a convenient and reliable tool for assessing muscle mass in patients undergoing dialysis [7,8]. The principle of the CKM is similar to that of the urinary creatinine excretion rate (uCER). Both modalities reflect the creatinine synthesis rate (CSR). Unlike healthy individuals, endogenously produced creatinine is predominantly removed by dialysis, and urinary excretion of creatinine is decreased or even absent in dialysis patients. Consequently, CKM requires collecting the dialysate effluent, urine, and both pre-dialysis and post-dialysis serum creatinine measures for calculation [7,9].

To simplify CKM measurement, Canaud et al. developed a modified creatinine index (mCI) equation recently [10]. The mCI is determined by age, sex, pre-dialysis creatinine, and single-pool Kt/V urea, all of which are measured regularly in clinical practice, making the conventional creatinine index simpler to calculate and easier to use. The clinical application of the mCI has received increasing attention. Recent studies have reported that the mCI is associated with clinical outcomes, such as bone fracture, cardiovascular events, and mortality, in patients undergoing hemodialysis (HD) [11–13]. The link between the mCI and adverse health outcomes remains unclear. It was hypothesized that the possible explanation may be muscle mass.

Muscle function, as another key factor in sarcopenia diagnosis, is not solely affected by muscle mass. Low muscle function and low muscle mass do not always occur in parallel [14–16]. It has been reported that plasma creatinine was correlated with muscle function, but not with muscle mass, in patients undergoing HD [16]. A previous study demonstrated uCER was associated with self-reported frailty [17], indicating that uCER may also reflect muscle function. Likewise, if the mCI could also reflect muscle function in patients undergoing HD, this might partly explain the association between the mCI and mortality because muscle function has been reported to be a strong predictor of death, whereas muscle mass is not [18]; muscle size may be less closely associated with mortality than functional status [19]. Actually, in the updated consensus paper drawn up by the European Working Group on Sarcopenia in Older People (EWGSOP2), low muscle strength overtook the role of low muscle mass, and probable sarcopenia is diagnosed when low muscle strength is detected, and this is adequate to trigger the assessment of causes and to initiate intervention [20].

Despite the growing interest, whether the mCI could identify persons with probable sarcopenia who may be at risk of adverse outcomes remains unknown. In this study, we primarily evaluated the relationship between the mCI and muscle strength, and find out the cutoff value for mCI to identify probable sarcopenia. Moreover, we verified the prognostic value of the mCI and compared its ability to predict mortality with that of the Modified Quantitative Subjective Global Assessment (MQSGA).

## Materials and methods

### Cross-sectional study

#### Study settings and participants

To evaluate the validity of the mCI in identifying probable sarcopenia and to determine its appropriate cutoff value. A single-center, cross-sectional study was conducted at the Blood Purification Center of a tertiary hospital from September 2020 to January 2021. The inclusion criteria were as follows: patients aged ≥18 years who were metabolically stable and undergoing HD treatment thrice per week for at least 8 weeks before enrollment. Meanwhile, patients who had contraindications for bioelectrical impedance analysis (BIA) (i.e., those with a pacemaker), had amputated limbs, had an acute infection, had cardiovascular events or hospitalization within 3 months before the study started, had malignancies, had severe edema, had cognitive impairment, and were wheelchair-bound or bed-ridden were excluded. The study was approved by the Research Ethics Committee of the hospital (no. 2020KY116). All participants provided written informed consent before inclusion.

#### Clinical, biological, and HD parameters

All the parameters were collected in one visit. The following data were recorded: age, sex, cause of end-stage kidney disease (ESKD), dialysis vintage, height; residual kidney function (RKF; defined as 24-h urine output >200 mL) [21]. Fasting blood was collected though the arteriovenous fistula or central venous catheter just before dialysis at the time of enrollment on the second dialysis day of the week. Biochemical parameters, including blood urea nitrogen [BUN], serum creatinine [SCr], triglyceride [TG], hypersensitive C-reactive protein [hs-CRP], albumin, total protein, total cholesterol [TCH], calcium, phosphorus, intact parathyroid hormone [iPTH], hemoglobin were measured using a fully automatic Biochemical Analyzer (Mindray BS800). Dialysis parameters were recorded at the same time. Single-pool Kt/V for urea, normalized protein equivalent of nitrogen appearance (nPNA) was measured according to the revalent equations [22]. The mCI was calculated by the following formula [10]:

Modified creatinine index(mg/kg/d)=16.21+1.12×[1 if male; 0 if female]−0.06 × age (years)−0.08 × single−pool Kt/V for urea+0.009 ×serum creatininebefore dialysis (μmol/L).

#### Assessment of nutritional status

MQSGA was used to evaluate the nutritional status. It consists of seven variables. Each component was assigned a score from 1 (normal) to 5 (very severe). The sum of all seven components in the malnutrition score lies between 7 (normal) and 35 (severely malnourished) [23]. It has been widely used in patients undergoing HD [24,25]. The MQSGA scores of all patients were performed by the same evaluator who had received the training of a professional nutritionist.

#### BIA measurement

BIA measurement was performed by Seca515 dual energy electrical impedance analyzer (Seca GmbH & Co., Hamburg, Germany) which is a multi-frequency bioelectrical impedance analyzer. The timing of the BIA measurement was set after the end of HD when the patients approached the estimated ideal weight, in order to eliminate the effects of excess fluid. We performed a BIA test 30 min after HD according to the National Kidney Foundation-Kidney Disease Outcomes Quality Initiative (NKF-DOQI) guidelines for the clinical application of BIA [26]. Our patients were instructed to eat only some small snacks in order to prevent hypoglycemia during the 4-h HD process and to perform at least 2 h of fasting before the BIA test. The patients were instructed to empty their bladder, remove their socks, and contact their hands and feet with an eight-point tactile electrode during the BIA test. Skeletal muscle index (SMI) was calculated using the following formula: SMI (kg/m^2^) = skeletal muscle mass (kg)/height^2^ (m^2^) [27]. Body mass index (BMI), fat tissue index (FTI), and waist circumference (WC) were also retrieved.

#### Muscle strength measurement

Muscle strength was measured by an electronic handgrip strength (HGS) meter (Guangdong Xiangshan Weighing Apparatus Group, China). The non-fistulation hand (or the dominant hand for patients with venous catheter) holds the meter tightly. HGS was measured twice before dialysis, and the highest value was used in the analysis. HGS was measured by the same operator at the same dialysis session with BIA measurements.

#### Physical activity level (PAL) measurements

The International Physical Activity Questionnaire (IPs) was used to assess the PAL. The reliability and validity was high [28]. The validity of the IPAQ Chinese version was verified in Chinese patients undergoing HD [29]. The questionnaire mainly evaluates the occupation, housework, transportation, and leisure physical activities of patients over the past week. The calculation of an individual’s weekly level of a certain physical activity is as follows: the MET assignment corresponding to the physical activity × weekly frequency (d/w) × time per day (min/day). The total PAL was the sum of the three levels (i.e., low, moderate, and high intensity) of physical activity.

#### Diagnosis of probable sarcopenia

Probable sarcopenia was diagnosed once low muscle strength (the most reliable marker of muscle function) was detected. Low muscle strength was defined as HGS <28 kg for men and <18 kg for women for Asian participants according to the updated consensus paper drew up by the Asian Working Group on Sarcooenia (AWGS) in 2020 [30].

### Longitudinal study

#### Study settings and participants

To evaluate the association between the mCI and mortality, we conducted a retrospective longitudinal cohort study using data from an independent cohort consisting of 218 patients who were 18 years or older and underwent regular HD therapy thrice per week for ≥3 months between March 2017 and June 2017. Patients who had malignancies, had an acute infection, had severe liver disease, had amputated limbs, were wheelchair-bound or bed-ridden, had hospitalization or cardiovascular events within 3 months before the study commenced were excluded. The study was approved by the Research Ethics Committee of the hospital (no. 2016LSKY). All participants provided written informed consent before inclusion.

#### Data collection

Data of the study participants were obtained: age; sex; underlying renal disease; HD vintage; prevalence of diabetes mellitus; history of cardiovascular events (acute coronary syndrome, cerebrovascular accident, hospitalization for congestive heart failure, and acute peripheral artery occlusion); alcohol and/or smoking habit; height; dry weight; MQSGA. All laboratory data, such as BUN, SCr, hs-CRP, albumin, TCH, hemoglobin, ferritin, were collected in one visit. Single-pool Kt/V for urea, nPNA and mCI were calculated according to the relevant equations.

#### Outcomes

The primary outcome was death from any cause. Baseline was defined as the date of the first measurement of laboratory data. We obtained vital status and date of death from the medical record system, and censored follow-up time at kidney transplantation, transfer, or the end of study follow-up (March 2022).

### Statistical analysis

The sample size needed to evaluate the ability of the mCI to predict sarcopenia was calculated using PASS11. The hypothesis of this study is that the area under the curve (AUC) of the mCI to predict sarcopenia was >0.5. A previous article showed that the AUC was approximately 0.69 [16]. The prevalence of sarcopenia was 20% [19], that is, the ratio between the size of the negative group and that of the positive group was 4:1. With an alpha level of 0.05 (one-sided) and a power of 90%, at least 125 patients should be enrolled in the study.

Normally distributed continuous variables, nonnormally distributed continuous variables, and categorical data are described as the mean ± standard deviation (SD), median and interquartile range, and percentage, respectively. The groups were compared using Student’s *t-*test, the Mann–Whitney *U*-test, or the chi-square test.

To examine the association of the mCI with low HGS in the cross-sectional study, univariate and multivariable logistic regression analyses were performed. The performance of the mCI in predicting low muscle strength was analyzed using receiver operating characteristic (ROC) curve analysis. Its sensitivity and specificity were also calculated. The appropriate cutoff points according to sex were determined using Youden’s method. The predictive ability of other parameters, including SCr, albumin, and phosphorus, was compared with that of the mCI using the DeLong test.

In the longitudinal study, participants were categorized into groups based on sex-specific mCI cutoff value, namely, the higher and lower groups. Unadjusted and multivariate-adjusted hazard ratios (HRs) and 95% CIs for death were estimated using Cox proportional regression models. The independent parameters that had *p* values <0.10 in the univariate analysis were deemed as covariates. Then, we performed multivariate analysis. Survival curves of study participants were described according to the Kaplan–Meier method to explore the impact of the mCI on survival. Differences between curves were evaluated using the log-rank test. We used Harrell’s C and net reclassification index (NRI) to compare the discrimination of the survival models [18,31]. We calculated the continuous NRI with 95% bootstrap CI to quantify the improvement in discrimination offered by adding mCI or MQAGA on the base model.

We conducted all analyses using SPSS software version 23.0 (IBM SPSS, Chicago, IL), R software version 4.1.3 (R Foundation for Statistical Computing, Vienna, Austria) and MedCalc Software version 11.4.2.0 (MedCalc, Mariakerke, Belgium). A 2-tailed *p* value <0.05 was considered statistically significant in all analyses.

## Results

### Cross-sectional results

#### Baseline characteristics of the study participants

The 346 participants enrolled in this study had a mean age of 58.17 ± 13.77 years; of the 346 patients, 38.4% were women. All patients had been treated with HD using high-flux polysulfone membrane dialyzers for a median of 52 months (interquartile range: 21–105.25). The primary causes of renal failure were chronic glomerulonephritis in 55.8% of the patients, diabetic nephropathy in 28%, polycystic kidney disease in 5.8%, hypertensive nephropathy in 4.9%, and other diseases in 5.5%. The patients included in this study underwent HD in a 4-h session thrice a week. The blood flow was 250–300 mL/min. The vascular access included arteriovenous fistula (96.5%) and venous catheter (3.5%). The average spKt/V was 1.54 ± 0.43.

Of the 346 patients on maintenance hemodialysis (MHD), 119 had low muscle strength, with a prevalence of 34.39%. Patients with low muscle strength were significantly older and had a higher prevalence of diabetic nephropathy than those with normal muscle strength. BUN, SCr, albumin, P, iPTH, mCI, SMI, BMI, HGS, and PAL were lower, whereas hs-CRP, MQSGA score, and FTI were higher in the low muscle strength group (Table 1).

**Table 1.** Clinical characteristics of the participants in the cross-sectional study.

Characteristics	Total (*N* = 346)	Low muscle strength group *N* = 119	Normal group *N* = 227	*p*
Age (years)	58.13 ± 13.77	67.26 ± 10.50	53.41 ± 12.86	<0.001
Sex				
Male, *n* (%)	213 (61.6%)	72 (60.5%)	141 (62.1%)	0.770
Dialysis vintage, months	52 (21,105.25)	58 (22,104)	47 (21,107)	0.448
ESRD primary cause				<0.001
Diabetic nephropathy, *n* (%)	97 (28.0%)	49 (41.2%)	48 (21.1%)	
Others, *n* (%)	249 (72.0%)	70 (58.8%)	179 (78.9%)	
RKF, n (%)	71 (20.5%)	28 (23.5%)	43 (18.9%)	0.316
Arteriovenous fistula, *n* (%)	334 (96.5%)	112 (94.1%)	222 (97.8%)	0.142
Kt/V for urea	1.54 ± 0.43	1.60 ± 0.39	1.51 ± 0.44	0.076
Biological parameters				
BUN (mmol/L)	22.06 ± 6.43	20.96 ± 8.06	22.64 ± 5.33	0.021
SCr (μmol/L)	860.22 ± 252.83	731.07 ± 187.51	927.93 ± 256.58	<0.001
TG (mmol/L)	1.49 (1.03,2.29)	1.57 (1.09,2.26)	1.44 (0.97,2.46)	0.434
Hs-CRP (g/L)	2.34 (1.07,5.29)	2.91 (1.48,7.45)	1.93 (0.98,4.57)	0.002
Albumin (g/L)	39.21 ± 3.25	38.11 ± 3.31	39.79 ± 3.08	<0.001
Total protein (g/L)	65.28 ± 5.16	64.82 ± 5.43	65.52 ± 5.00	0.229
Hemoglobin (g/L)	111.42 ± 15.41	110.08 ± 16.43	112.12 ± 14.83	0.242
TCH (mmol/L)	4.12 ± 1.13	4.19 ± 1.15	4.08 ± 1.12	0.412
iPTH (pg/mL)	315.85 (152.88,548.10)	247.70 (122.90,513.30)	348.10 (196.60,559.50)	0.028
Calcium (mmol/L)	2.28 ± 0.19	2.26 ± 0.21	2.30 ± 0.18	0.090
Phosphorus (mmol/L)	1.83 ± 0.50	1.65 ± 0.42	1.92 ± 0.51	<0.001
mCI (mg/kg/d)	21.03 ± 2.91	19.30 ± 2.06	21.93 ± 2.89	<0.001
nPNA (g/kg/d)	1.12 ± 0.31	1.10 ± 0.42	1.13 ± 0.24	0.399
MQSGA score	10.97 ± 2.21	11.74 ± 2.43	10.57 ± 1.98	<0.001
BIA				
SMI (kg/m^2^)	7.15 ± 1.60	6.26 ± 1.32	7.61 ± 1.53	<0.001
BMI (kg/m^2^)	22.60 ± 3.46	22.13 ± 2.78	22.84 ± 3.75	0.047
Waist circumstance (cm)	0.85 ± 0.12	0.85 ± 0.13	0.84 ± 0.12	0.441
FTI (kg/m^2^)	6.93 ± 2.75	7.72 ± 2.56	6.52 ± 2.76	<0.001
HGS (kg)	27.65 ± 9.62	19.78 ± 6.23	31.79 ± 8.44	<0.001
PAL (MET)	1224.32 ± 811.93	950.27 ± 681.51	1137.98 ± 838.79	<0.001

Values for continuous variables are given as means ± standard deviations or medians and interquartile ranges. Categorical variables are expressed as numbers (%). RKF: residual kidney function; BUN: blood urea nitrogen; SCr: serum creatinine; TG: triglyceride; hs-CRP: high-sensitivity C-reactive protein; TCH: total cholesterol; iPTH: intact parathyroid hormone; mCI: modified creatinine index; nPNA: normalized protein equivalent of nitrogen appearance; MQSGA: Modified Quantitative Subjective Global Assessment; BIA: bioimpedance analysis; SMI: skeletal muscle mass index; BMI, body mass index; FTI: fat tissue index; HGS: handgrip strength; PAL: physical activity level; MET: metabolic equivalent.

#### Association of the mCI with low muscle strength

The odds ratios for the associations of the mCI with muscle strength are presented in Table 2. Univariate analysis of the association showed that the percentage of participants who had low muscle strength decreased by increasing the mCI value. The association remained significant after adjusting for the confounders (Model 1). After further adjusting for SMI, the association remained significant, though the relevance was somewhat attenuated (Model 2).

**Table 2.** Association of mCI with low muscle strength.

	OR [95%CI]	*p* Value
Unadjusted	0.66 [0.59, 0.74]	<0.001
Model 1	0.71 [0.61, 0.83]	<0.001
Model 2	0.75 [0.64, 0.88]	<0.001

Model 1: adjusted for age, sex, dialysis vintage and presence of diabetic nephropathy, BUN, serum albumin, hs-CRP, serum phosphorus, iPTH, MQSGA score, BMI, FTI, PAL.

Model 2: adjusted for all variables in Model 1 and additionally SMI.

#### Performance of the mCI in identifying probable sarcopenia

**Figure 1.**
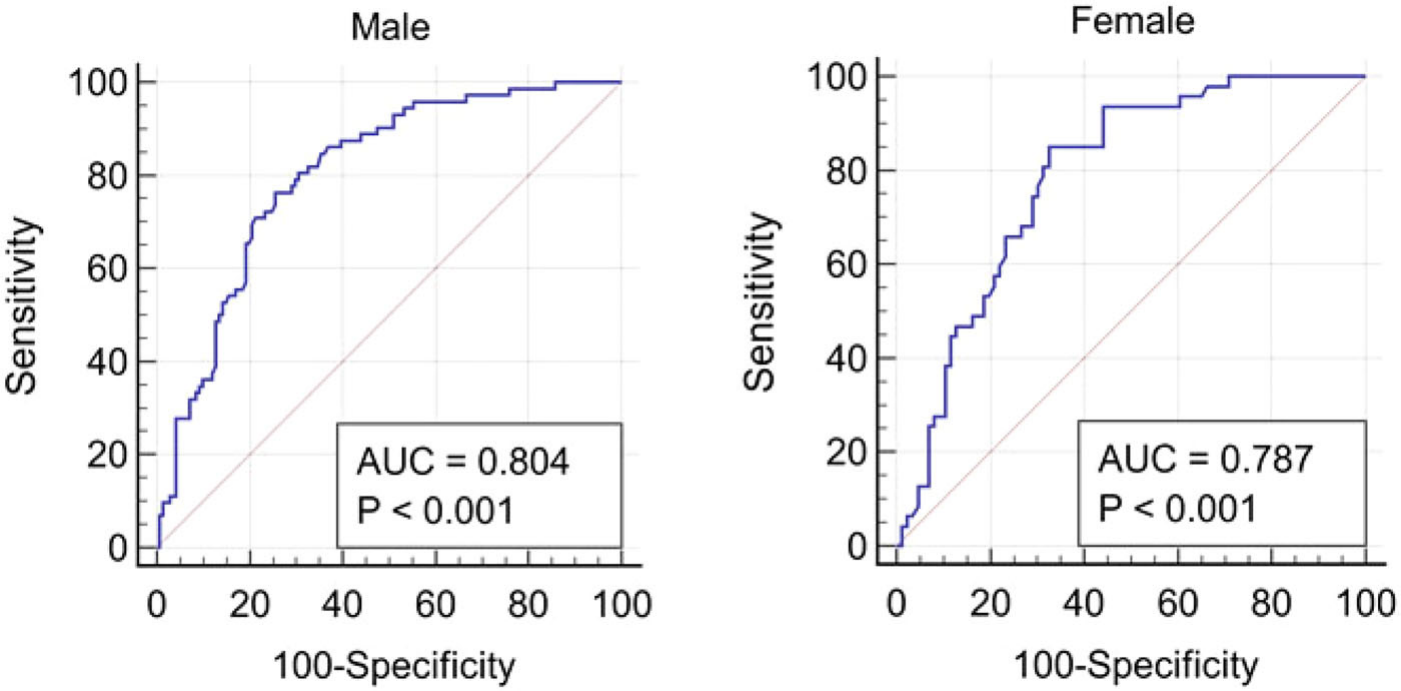
Receiver operating characteristic (ROC) curves of the modified creatinine index for predicting probable sarcopenia in male and female hemodialysis patients.

Probable sarcopenia was diagnosed once low muscle strength was detected. The results showed that the AUC of the mCI for predicting probable sarcopenia was 0.774 (95% CI, 0.724–0.823; *p* < 0.001), which was considered excellent. Considering the impact of sex on the results, we also performed a separate analysis by sex. The AUC of the mCI to predict probable sarcopenia was 0.804 for men (95% CI, 0.744–0.863; *p* < 0.001) and 0.787 for women (95% CI, 0.711–0.864; *p* < 0.001) (Figure 1). The optimal mCI cutoff values of ≤21.07 for men and ≤19.57 for women yielded a sensitivity of 76.39% and a specificity of 74.47% for men, a sensitivity of 85.11% and a specificity of 67.44% for women, respectively.

**Figure 2.**
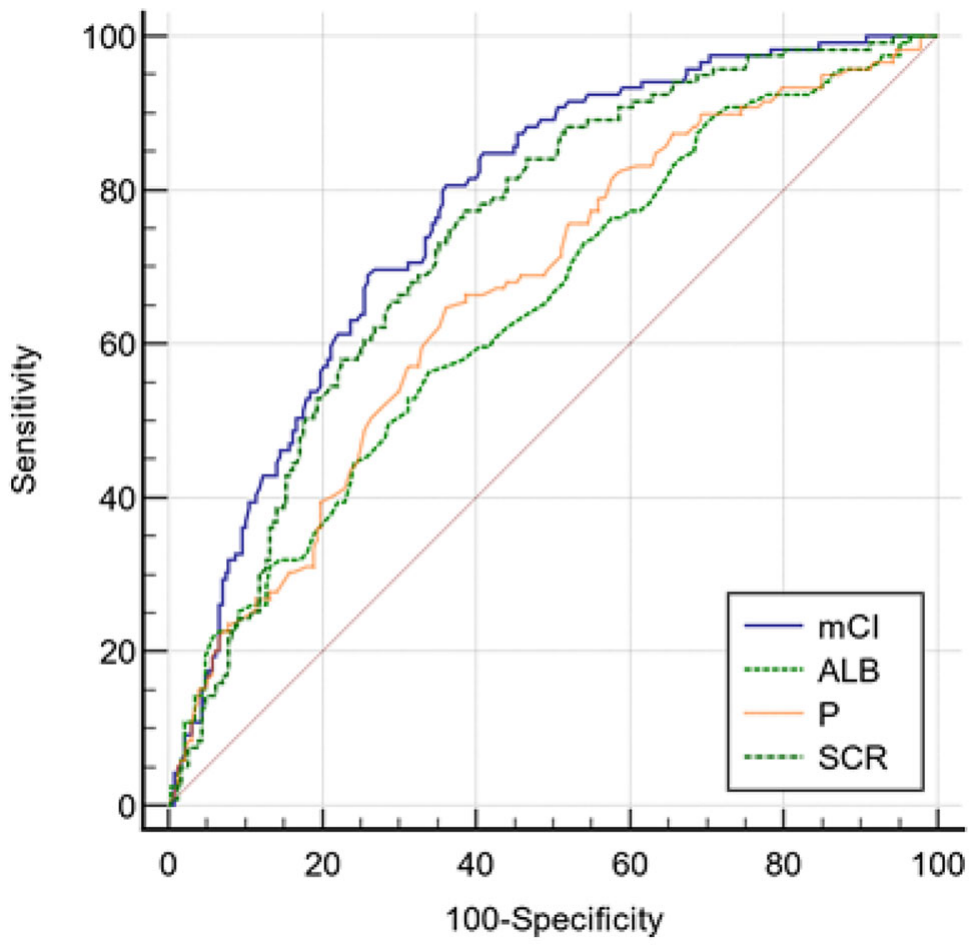
Receiver operating characteristic (ROC) curves of the modified creatinine index (mCI), albumin (ALB), phosphorus (P) and serum creatinine (SCR) for predicting probable sarcopenia in hemodialysis patients.

The performance of other parameters, including albumin, phosphorus, and SCr, in predicting probable sarcopenia, as represented by the AUC, is also shown in Table 3, and they were compared with that of mCI. The results showed that the mCI was better than albumin, phosphorus, and SCr (Table 3 and Figure 2).

**Table 3.** Comparison of areas under the ROC curve for predicting probable sarcopenia.

Variables	AUC	95%CI	*p* Value
mCI	0.774	0.724–0.823	Ref.
Albumin	0.647	0.594–0.697	<0.001
Phosphorus	0.666	0.614–0.716	<0.001
SCr	0.740	0.690–0.785	<0.001

AUC: area under the roc curve; mCI: modified creatinine index; SCr: serum creatinine.

### Longitudinal results

#### Baseline characteristics of the participants of retrospective longitudinal cohort study

All data were stratified according to sex-specific cutoff values of the mCI. Patients with higher mCI values were more likely to be younger, with lower prevalence rates of diabetes mellitus, higher BUN, SCr, albumin, nPNA, and lower hs-CRP (Table 4).

**Table 4.** Clinical characteristics of the participants in each group stratified according to sex-specific cutoff values of mCI in the longitudinal cohort study.

Characteristics	Lower mCI (*n* = 86)	Higher mCI (*n* = 132)	*p*
Age (years)	70.61 ± 10.61	55.01 ± 11.96	<0.001
Gender			0.700
Male, *n* (%)	55 (64.0)	81 (61.4)	
Female, *n* (%)	31 (36.0)	51 (38.6)	
Dialysis vintage, months	55.00 (29.75–98.00)	67.50 (36.25–107.75)	0.112
ESRD primary cause			
Diabetic nephropathy, *n* (%)	29 (33.7)	24 (18.2)	0.009
Others, *n* (%)	57 (66.3)	108 (81.8)	
Comorbid conditions			
History of cardiovascular events	15 (17.4%)	12 (9.1%)	0.067
Diabetes, *n* (%)	36 (41.9%)	32 (24.2%)	0.006
Smoking (%)	11 (12.8)	19 (14.4)	0.737
Alcohol (%)	12 (14%)	9 (6.9%)	0.084
BMI (kg/m^2^)	21.65 ± 3.09	21.39 ± 2.63	0.494
Kt/V for urea	1.66 ± 0.31	1.64 ± 0.33	0.773
Biological parameters			
BUN (mmol/L)	20.32 ± 4.32	23.87 ± 5.26	<0.001
SCr (μmol/L)	707.547 ± 121.690	1020.00 ± 157.77	<0.001
hs-CRP (g/L)	3.29 (1.18-5.63)	1.86 (1.04-3.36)	0.003
Albumin (g/L)	38.59 ± 2.06	39.66 ± 2.05	<0.001
TCH (mmol/L)	4.06 ± 0.93	4.04 ± 0.92	0.856
Hemoglobin (g/L)	102.38 ± 13.83	104.89 ± 12.53	0.167
Ferritin (ng/ml)	93.40 (44.68, 205.13)	111.45 (48.75, 244.18)	0.256
mCI (mg/kg/d)	18.93 ± 1.45	22.65 ± 1.87	<0.001
nPNA (g/kg/d)	1.08 ± 0.21	1.24 ± 0.29	<0.001

Values for continuous variables are given as the means ± standard deviations or medians and interquartile ranges. Categorical variables are expressed as numbers (%). BMI: body mass index; BUN: blood urea nitrogen; SCr: serum creatinine; hs-CRP: high-sensitivity C-reactive protein; TCH: total cholesterol; mCI: modified creatinine index; nPNA: normalized protein equivalent of nitrogen appearance.

#### Association of low mCI and MQSGA score with mortality

During a median observational period of 5 years (interquartile range: 41.5–60.0 months), 42 deaths (32 men and 10 women) occurred; these included 19 (45.2%), 12 (28.6%), 3 (7.1%), and 8 (19.0%) deaths due to cardiovascular disease, infection, malignancies, and other causes, respectively.

**Figure 3.**
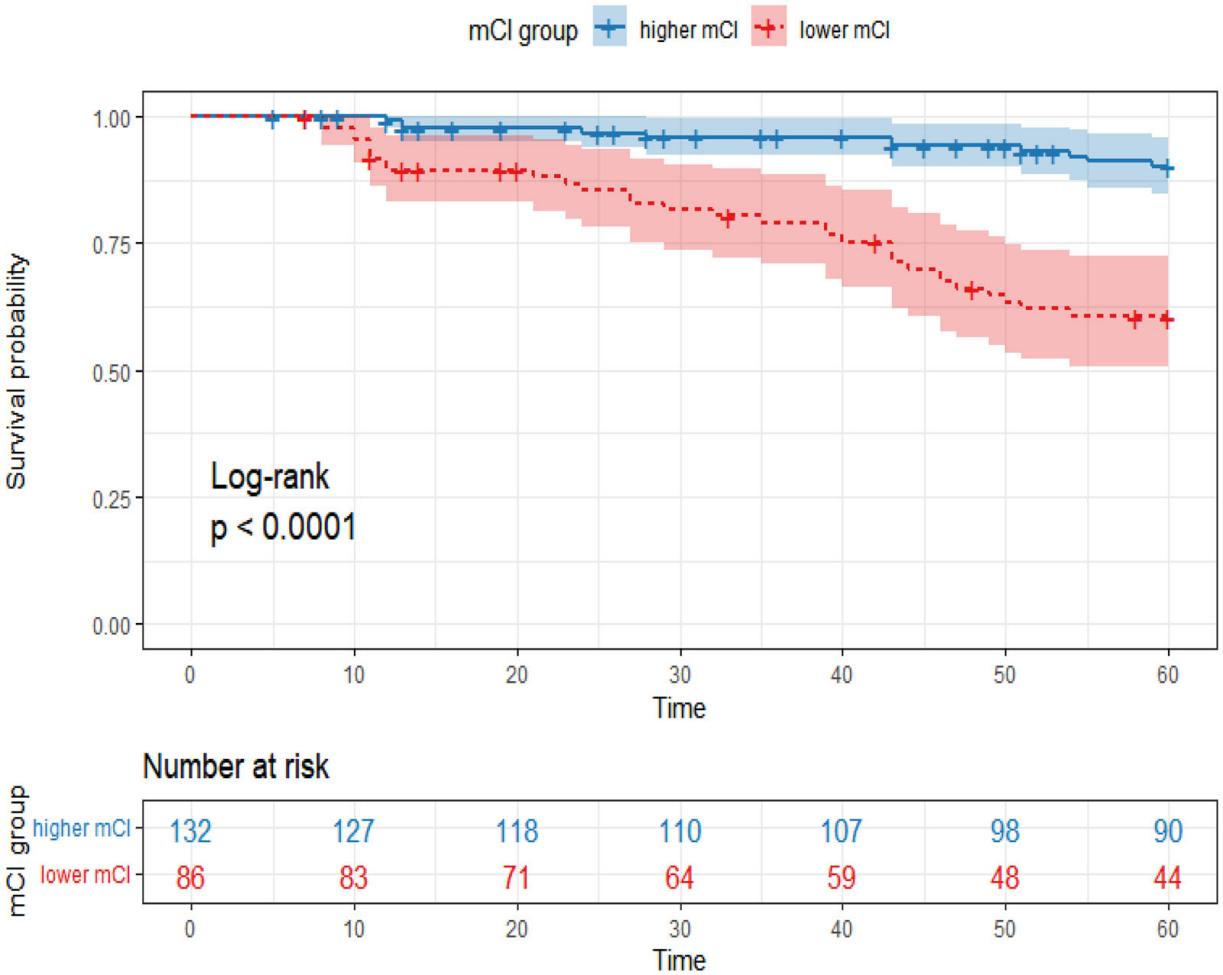
Kaplan–Meier curves for the survival probability in each group stratified by sex-specific modified creatinine index (mCI).

The Kaplan–Meier curves showed significantly higher mortality in the lower mCI group than in the higher mCI group (log-rank test, *p* < 0.001) (Figure 3). The results of the unadjusted and adjusted Cox proportional hazards models are shown in Tables 5 and 6. In both the unadjusted and multivariate-adjusted models, patients with lower mCI values were associated with higher adjusted HRs for mortality than those with higher mCI values. After adjusting for potential covariates, the participants in the lower mCI group still had a higher risk of death for all causes (HR, 2.51; 95% CI, 1.16–5.41; *p* = 0.019). However, after adjusting for age and sex, no association was observed between the MQSGA and mortality.

**Table 5.** Univariate Cox analysis of potential factors associated with all-cause mortality^a^.

Variables	Unadjusted hazard ratio [95% CI]	*p* Value
Age (years)	1.08 [1.05, 1.11]	<0.001
Sex (male)	2.31 [1.13, 4.69]	0.021
Dialysis vintage (months)	0.993 [0.987, 1.000]	0.051
Diabetes	2.27 [1.24, 4.15]	0.008
History of cardiovascular events	2.56 [1.23, 5.22]	0.010
Albumin (g/L)	0.82 [0.72, 0.94]	0.004
Hs-CRP (g/L)	1.04 [1.02, 1.06]	<0.001
MQSGA	1.113 [1.010, 1.227]	0.030
Low mCI	5.01 [2.52, 9.94]	<0.001

^a^ Only variables with *p* values <0.10 in univariate analysis were shown.

hs-CRP: high-sensitivity C-reactive protein; MQSGA: Modified Quantitative Subjective Global Assessment; mCI: modified creatinine index.

**Table 6.** Multivariate Cox analysis of low mCI and MQSGA for all-cause mortality.

Variables	Hazard ratio [95% CI]	*p* Value
mCI ( versus high mCI group)		
Unadjusted	5.01 [2.52, 9.94]	<0.001
Model 1	2.86 [1.32, 6.20]	0.008
Model 2	2.67 [1.22, 5.85]	0.014
Model 3	2.51 [1.16, 5.41]	0.019
MQSGA (per 1-unit increase)		
Unadjusted	1.113 [1.010, 1.227]	0.030
Model 1	0.971 [0.865, 1.091]	0.621
Model 2	1.004 [0.888, 1.136]	0.945
Model 3	1.024 [0.902, 1.163]	0.715

Model 1: adjusted for age, sex.

Model 2: adjusted for age, sex, dialysis vintage, diabetes, history of cardiovascular events.

Model 3: adjusted for age, sex, dialysis vintage, diabetes, history of cardiovascular events, albumin, hs-CRP.

#### Model discrimination in predicting all-cause mortality

When comparing with a traditional risk model accounting for classical risk indictors (age, sex, dialysis vintage, diabetes, history of cardiovascular events, albumin, and hs-CRP), adding the lower mCI significantly improved the C-index from 0.785 to 0.805 (*p* = 0.026) and improved the continuous NRI (38.6%; 95% CI, 5.8%–71.4%; *p* = 0.021) (Table 7), while adding MQSGA did not.

**Table 7.** Predictive accuracies of mCI and MQSGA for all-cause mortality.

Models	C-index [95% CI]	*p* Value	Continuous NRI (%) [95% CI]	*p* Value
Base model^a^	0.785 [0.708, 0.862]	Ref.		Ref.
+ low mCI	0.805 [0.733, 0.877]	0.026	38.6 [5.8, 71.4]	0.021
+ MQSGA	0.784 [0.706, 0.861]	0.892	−7.3 [−40.8, 26.3]	0.672

^a^Containing age, sex, dialysis vintage, diabetes, history of cardiovascular events, albumin, hs-CRP.

mCI: modified creatinine index; MQSGA: Modified Quantitative Subjective Global Assessment; NRI: net reclassification index.

## Discussion

This study had three important findings: (1) the mCI was significantly positively associated with muscle strength in patients undergoing HD, highlighting the mCI as a practical tool to screen for sarcopenia. (2) The optimal cutoff values of the mCI were determined. (3) The mCI classified by the cutoff value was useful to stratify risks of all-cause mortality, and the mCI was preferable to the MQSGA for predicting death.

Muscle strength is presently the most reliable measure of muscle function and is an indicator of probable sarcopenia. Our research showed that the mCI was independently associated with muscle strength. Likewise, the CSR has also been found to be associated with muscle strength and frailty [17,32]. And the uCER was an indicator of physical performance and function [33,34].

Of note, our study showed that the association of mCI with muscle strength was significant, even after further adjusting for SMI, indicating that mCI reflects muscle function. Our view is further supported by a cohort study including patients with CKD stages 1 through 5, it was found that the creatinine generation rate per kg of fat-free mass was lower among patients with lower renal function. This might be explained by altered creatine metabolism, leading to a lower creatinine generation rate and poorer quality and thus low muscle function [35]. Second, a murine study found a strong linear correlation between the CSR and myofibrillar protein mass in rat muscle, and CSR was a valid indicator of contractile muscle mass [36]. Third, a recent study conducted involving a Japanese population consisting of older community residents found that the creatinine-to-cystatin C ratio was inversely associated with the cross-sectional areas of fat-rich muscles and positively associated with that of muscle fiber-rich muscles. The creatinine-to-cystatin C ratio showed a significant association with the mean attenuation value of skeletal muscle, a representative measure of myosteatosis, independent of its cross-sectional area. This indicated that the creatinine-to-cystatin C ratio could serve as a convenient marker of muscle quantity and quality [37]. Fourth, Wilson et al. reported that a lower uCER was an independent predictor of death in patients with CKD even after adjusting for FFM, while FFM was not. The possible explanation was that the uCER might capture information about muscle quality that was independent of muscle mass [35]. Generally, it could be speculated that the mCI might particularly capture information on functional and metabolic active muscle mass. The generation of creatinine from the non-enzymatic conversion of creatine and creatine phosphate in muscle guarantees that it is insensitive to intramuscular fat and thereby provides a direct reflection of the ‘active muscle mass’, and thus reflects muscle function.

Our results showed that the optimal mCI cutoff value for identifying probable sarcopenia was ≤21.07 for men and ≤19.57 for women, with 76.39% sensitivity and 74.47% specificity for men and 85.11% sensitivity and 67.44% specificity for women. The predictive ability of the mCI was considered to be acceptable-to-excellent, and its performance was better than that of other parameters, including albumin, phosphorus, and SCr. To identify possible cases in time, the SARC-F was recommended by the EWSOP to screen patients at risk of sarcopenia. This is a 5-item questionnaire that is self-reported by patients. The sensitivity of the SARC-F to predict probable sarcopenia was reported to be 33.7–50%, with a specificity of 93.7–85.8% [38]. Although with excellent specificity, its low-to-moderate sensitivity may indicate the low capacity of a screen tool to detect subjects at a high risk of developing sarcopenia [20]. In contrast, the high sensitivity of the mCI to screen for probable sarcopenia lays the foundation for clinical screening of probable sarcopenia in patients undergoing HD. Furthermore, the mCI is easy to apply and monitor in clinical practice as the measurement does not need any equipment, and only routinely gathered data that are already present in electronic health records are needed.

The objective of case finding is to identify persons at a high risk of adverse clinical outcomes. Our results showed that the mCI could predict survival. Moreover, adding mCI to the baseline evaluation model consisting of classical risk factors significantly improved the predictably of all-cause mortality, as observed in the discrimination analysis model. Thus, the mCI classified by the cutoff value was useful in stratifying risks of all-cause mortality and was preferred over the MQSGA for predicting death in patients undergoing HD. These results validated the clinical value of the mCI as a simple tool to detect persons at risk of adverse outcomes from probable sarcopenia. A few studies involving patients undergoing HD also reported that the mCI was significantly associated with greater survival, and those studies analyzed data using arbitrary cutoff points for high mCI derived from their cohorts and the cutoff points were not always consistent. Differently from them, we determined the optimal cutoff value of the mCI to identify probable sarcopenia and validated the prognostic value, which was important and practical in clinical use. To the best of our knowledge, this is the first study. Our research provides a convenient and adequate method that can be easily adopted clinically to identify persons with probable sarcopenia, who may be amenable to treatment.

However, there are limitations to this study. First, SCr may have been influenced by the dietary intake of protein and dialysis dose [39,40]. However, the mCI considers the effects of dialysis dose by incorporating the Kt/V into the calculation formula. Furthermore, data about nPNA, a marker of dietary protein intake, were also collected and analyzed in this study. It showed no significant effect on our results. Second, we excluded patients with the highest risk of sarcopenia, such as those in bed or wheelchair. Therefore, more studies are required to extrapolate the results to these patients. Third, we used the BIA technique, not dual-energy X-ray absorptiometry, to measure muscle mass. The accuracy of the muscle mass measurement using BIA in patients undergoing HD has been confirmed and applied in multiple studies [16,18,41–46], and BIA was recommended to be used to assess body composition according to the NKF-DOQI guidelines [26]. Finally, because this study involved Chinese patients undergoing HD, the findings may, therefore, not apply to other countries and cultures.

## Conclusion

The mCI is a predictor of muscle strength and survival in hemodialysis patients. It is a simple, quick, valid and practical tool for identifying probable sarcopenia, and is preferable to the MQSGA for predicting death. Assessment of mCI could provide additional predictive and prognostic information to sarcopenia.

## Data Availability

The datasets analyzed during the current study are available from the corresponding author on reasonable request.
